# Reduced Gray Matter Volume in Orbitofrontal Cortex Across Schizophrenia, Major Depressive Disorder, and Bipolar Disorder: A Comparative Imaging Study

**DOI:** 10.3389/fnins.2022.919272

**Published:** 2022-06-10

**Authors:** Yongfeng Yang, Xue Li, Yue Cui, Kang Liu, Haoyang Qu, Yanli Lu, Wenqiang Li, Luwen Zhang, Yan Zhang, Jinggui Song, Luxian Lv

**Affiliations:** ^1^Department of Psychiatry, Henan Mental Hospital, Second Affiliated Hospital of Xinxiang Medical University, Xinxiang, China; ^2^Henan Key Lab of Biological Psychiatry, Xinxiang Medical University, Xinxiang, China; ^3^International Joint Research Laboratory for Psychiatry and Neuroscience of Henan, Xinxiang, China; ^4^Brainnetome Center and Institute of Automation, Chinese Academy of Sciences, Beijing, China; ^5^National Laboratory of Pattern Recognition, Institute of Automation, Chinese Academy of Sciences, Beijing, China; ^6^University of Chinese Academy of Sciences, Beijing, China; ^7^Department of Psychiatry, The Second Clinic College of Xinxiang Medical University, Xinxiang, China

**Keywords:** schizophrenia, major depressive disorder, bipolar disorder, gray matter volume (GMV), psychopathology

## Abstract

Schizophrenia (SZ), major depressive disorder (MDD), and bipolar disorder (BD) are severe psychiatric disorders and share common characteristics not only in clinical symptoms but also in neuroimaging. The purpose of this study was to examine common and specific neuroanatomical features in individuals with these three psychiatric conditions. In this study, 70 patients with SZ, 85 patients with MDD, 42 patients with BD, and 95 healthy controls (HCs) were recruited. Voxel-based morphometry (VBM) analysis was used to explore brain imaging characteristics. Psychopathology was assessed using the Beck Depression Inventory (BDI), the Beck Anxiety Inventory (BAI), the Young Mania Rating Scale (YMRS), and the Positive and Negative Syndrome Scale (PANSS). Cognition was assessed using the digit symbol substitution test (DSST), forward-digital span (DS), backward-DS, and semantic fluency. Common reduced gray matter volume (GMV) in the orbitofrontal cortex (OFC) region was found across the SZ, MDD, and BD. Specific reduced GMV of brain regions was also found. For patients with SZ, we found reduced GMV in the frontal lobe, temporal pole, occipital lobe, thalamus, hippocampus, and cerebellum. For patients with MDD, we found reduced GMV in the frontal and temporal lobes, insular cortex, and occipital regions. Patients with BD had reduced GMV in the medial OFC, inferior temporal and fusiform regions, insular cortex, hippocampus, and cerebellum. Furthermore, the OFC GMV was correlated with processing speed as assessed with the DSST across four groups (*r* = 0.17, *p* = 0.004) and correlated with the PANSS positive symptoms sub-score in patients with SZ (*r* = − 0.27, *p* = 0.026). In conclusion, common OFC alterations in SZ, MDD, and BD provided evidence that this region dysregulation may play a critical role in the pathophysiology of these three psychiatric disorders.

## Introduction

Schizophrenia (SZ), major depressive disorder (MDD), and bipolar disorder (BD) are highly complex psychiatric disorders for which the diagnosis primarily depends on the patient’s clinical symptoms and the psychiatrist’s experiences. The literature has shown that these psychiatric disorders share some common genetic vulnerability and clinical symptoms (Wang et al., 2017; Cross-Disorder Group of the Psychiatric Genomics Consortium, 2019).

Previous research using voxel-based morphometry (VBM) found that decreased gray matter (GM) volume in the right inferior frontal gyrus was a common abnormality feature in both MDD and BD (Cai et al., 2015). Atrophy or decreased GM volume (GMV) was found in the subgenual anterior cingulate cortex (ACC) in MDD and in the subcallosal ACC (Niida et al., 2014, 2019) and bilateral fronto-insular cortex (Bora et al., 2012) in BD. Further analysis revealed that patients with both MDD and BD showed decreased GMV in the bilateral insula cortex (Wise et al., 2016), left ACC, and right hippocampus (Chen et al., 2018). Patients with SZ showed some evidence of GMV reduction in the prefrontal and temporal cortex (Yuksel et al., 2012; Nenadic et al., 2015; Knochel et al., 2016), bilateral insular cortex (Niida et al., 2019), thalamus, hippocampus, striatum, and cerebellum (Watson et al., 2012; Nenadic et al., 2015). In addition, it was found that patients with both SZ and BD had some common GMV reduction in the superior temporal gyrus (STG) and inferior parietal lobule (Cui et al., 2011).

A meta-analysis found GM loss in the bilateral insula and dorsal ACC across SZ, BD, depression, addiction, anxiety, and obsessive-compulsive disorder (Goodkind et al., 2015). Chang et al. (2018) reported that the SZ, MDD, and BD shared reduced GMV in 87.9% of the whole brain regional volume. In addition, previous studies found functional abnormalities of the insula in MDD (Wang et al., 2018; Wang J. et al., 2020; Wang L. et al., 2020).

However, the common and specific GMV studies of SZ, MDD, and BD are scarce and inconsistent. Therefore, in the present study, we hypothesize that SZ, MDD, and BD may have common specific characteristics that can be explored by brain structure imaging. Further, this study may provide objective image markers for the diagnosis and differential diagnosis of these three psychiatric disorders. Thus, we used VBM analysis to explore possible common and specific changes in neuroimaging features in patients with SZ, MDD, and BD.

## Materials and Methods

### Participants

The participants included four groups of subjects: SZ (*n* = 70), MDD (*n* = 85), BD (*n* = 42), and healthy controls (HCs, *n* = 95). The study was conducted between March 2013 and October 2017 at the Second Affiliated Hospital of Xinxiang Medical University, China. The study was approved by the Ethics Committee of the Second Affiliated Hospital of Xinxiang Medical University. All participants provided written informed consent. All participants were between 18 and 55 years old, Han Chinese in origin, and right-handed. Patients with SZ, MDD, and BD were independently diagnosed by two experienced psychiatrists using the Structured Clinical Interview for Diagnostic and Statistical Manual of Mental Disorders (DSM)-IV Axis I Disorders. Exclusion criteria were the following: heart, kidney, or liver disease; other mental disorders; and the presence of implanted metal frames or electronic devices preventing Magnetic Resonance Imaging (MRI) scanning. Symptoms were measured using the Beck Anxiety Inventory (BAI) (Beck et al., 1988) and Beck Depression Inventory (BDI) (Beck et al., 1961) for patients with MDD, the BDI, BAI, and Young Mania Rating Scale (YMRS) (Young et al., 1978) for patients with BD, and the Positive and Negative Syndrome Scale (PANSS) (Kay et al., 1987) for patients with SZ. Patients with BD were further diagnosed with depression (*n* = 15), mania (*n* = 25), and stable mood (*n* = 2). Cognitive function tests were carried out using the digit symbol substitution test (DSST), forward-digital span (DS), backward-DS, and semantic fluency. None of the HCs had a personal history of psychotic illness or a family history of psychosis in their first-, second-, or third-degree relatives. HCs were excluded if one of the following were present: (1) history of head injury, (2) the presence of a major and unstable physical illness, (3) heart, kidney, or liver disease, (4) pregnant or breast-feeding women, or planning pregnancy, and (5) the presence of implanted metal frames or electronic devices preventing MRI scanning.

### Data Acquisition and Pre-processing

All participants underwent T1-weighted imaging using a 3.0 Tesla Siemens Scanner (Siemens, Verio, Germany). Acquisition parameters for T1-weighted scans were as follows: repetition time = 2,530 ms, echo time = 2.43 ms, inversion time = 1,100 ms, flip angle = 7°, matrix size = 256 × 256 × 192, and voxel size = 1 × 1 × 1 mm. Foam pads and earplugs were used to reduce head motion and scanner noise.

All T1-weighted images were processed using Statistical Parametric Mapping (SPM12, Wellcome Department of Imaging Neuroscience, London, United Kingdom)^1^ and the Computational Anatomy Toolbox (CAT12). Briefly, the images were bias-corrected and segmented into different tissues, such as GM, white matter, and cerebrospinal fluid images. The tissue images were then spatially normalized and resampled to a resolution of 1.5 × 1.5 × 1.5 mm^3^. To preserve regional volumetric information, the images were modulated by the Jacobian determinants of the deformations during the warping. Finally, 6-mm full width at half maximum Gaussian kernel smoothing was performed to generate the voxel-based GMVs for each subject for the subsequent statistical analysis.

### Statistical Analysis

The demographic characteristics of the groups were compared using analysis of variance and χ^2^-tests. MRI data were analyzed using the CAT12. Group differences in regional GMVs were investigated by comparing the pre-processed GM images from each pair of groups using a general linear model with age, gender, and total intracranial volume (TIV) as covariates. We performed AlphaSim correction with a threshold of *p* < 0.01 for multiple comparisons. The correction was achieved with a voxelwise threshold of *p* < 0.005 and a minimum cluster extent of 298 voxels. Identification of overlapping regions across mental disorders was performed on the basis of regions showing consistent GM changes after multiple corrections. No statistical method was used to identify overlapping regions. Pearson’s correlations were used to assess (i) relationships between GMVs in overlapping regions and digit symbol scores across all groups and (ii) relationships between GMVs and symptom severity as measured by BDI, BAI, and PANSS scales in MDD, BD, and SZ. Partial correlation analysis was performed using age, sex, TIV, and the group as confounding variables.

## Results

### Sociodemographic and Clinical Characteristics

Sociodemographic and clinical data are presented in Table 1. No statistically significant differences in age or sex were noted between each pair of two groups except that patients with SZ were younger than patients with BD (SZ vs. BD, *t* = − 3.35, *p* = 0.001) and patients with MDD (SZ vs. MDD, *t* = − 3.27, *p* = 0.001). Significant differences were also observed in the duration of illness, age of onset, and medication status across patient groups.

**TABLE 1:** Demographic characteristics of the participants.

	MDD (*n* = 85)	BD (*n* = 42)	SZ (*n* = 70)	HC (*n* = 95)	*F*/χ^2^-values	*P*-values
Male (%)	40.0	54.8	44.3	49.5	1.09	0.350
Age (years)	32.44 (8.79)	32.88 (8.79)	28.40 (4.92)*^a^*	30.21 (6.85)	5.15	0.002*
Education (years)	11.5 (3.60) (*n* = 84)	10.5 (3.83) (*n* = 40)	11.48 (2.76) (*n* = 64)	13.80 (2.88) (*n* = 94)	13.89	<0.001
Age of onset (years)	29.18 (9.32) (*n* = 84)	26.25 (8.88) (*n* = 40)	24.54 (4.86) (*n* = 64)	N/A	6.61	0.002
Medication (Yes/No)	66/19	37/5	44/26	N/A	9.55	0.008
Antipsychotics (Yes)	5	24	44	N/A	62.66	<0.001
Antidepressants (Yes)	64	15	0	N/A	91.03	<0.001
Mood stabilizer	1	24	0	N/A	95.25	<0.001
Duration of illness (months)	40.81 (55.87) (*n* = 84)	77.10 (77.16) (*n* = 40)	43.26 (43.28) (*n* = 64)	N/A	5.89	0.003
Digit symbol	48.56 (13.40) (*n* = 81)	47.53 (15.25) (*n* = 40)	38.18 (10.47) (*n* = 57)	62.31 (11.92) (*n* = 94)	11.6	<0.001
BDI	18.60 (7.66) (*n* = 68)	9.95 (11.71) (*n* = 40)	N/A	N/A	21.55	<0.001
BAI	42.83 (11.41) (*n* = 48)	31.85 (13.75) (*n* = 39)	N/A	N/A	16.59	<0.001
PANSS total	N/A	N/A	78.5 (18.15)	N/A	N/A	N/A
PANSS positive	N/A	N/A	22.74 (3.75)	N/A	N/A	N/A
PANSS negative	N/A	N/A	19.86 (5.29)	N/A	N/A	N/A

*Values are mean (SD) unless otherwise indicated.*

*BAI, Beck Anxiety Inventory; BD, bipolar disorder; BDI, Beck Depression Inventory; HC, healthy control; MDD, major depressive disorder; N/A, not applicable; PANSS, Positive and Negative Syndrome Scale; SZ, schizophrenia.*

*SZ had significantly younger age in years than BD and MDD. No significant differences were found in age or gender between three mental disorders (MDD, BD, and SZ) and HC.

### Voxel-Based Morphometry Comparisons

**FIGURE 1:**
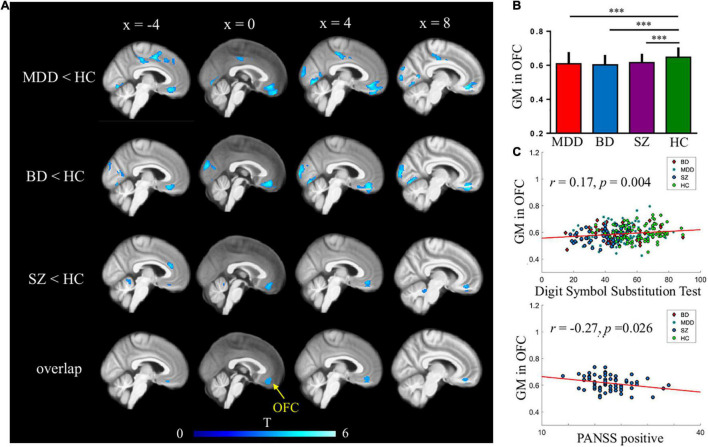
Common gray matter (GM) deficits in major depressive disorder (MDD), bipolar disorder (BD), and schizophrenia (SZ). **(A,B)** Reduced GMVs were found in the medial orbitofrontal cortex (OFC) across three mental disorders. AFNI’s AlphaSim was used for multiple comparisons corrections; **(C)** GMVs in medial OFC were correlated with digit symbol substitution test across four groups and PANSS positive scales in patients with SZ.

Patients with SZ, MDD, and BD had significantly reduced regional GMVs when compared to HCs. The common GMV was reduced in the orbitofrontal cortex (OFC) region of patients with SZ, MDD, and BD. Specifically, patients with SZ had reduced GM in the frontal lobe, temporal lobe, occipital lobe, thalamus, hippocampus, and cerebellum. For patients with MDD, the differences were most pronounced in the frontal and temporal lobes, insular cortex, and occipital regions. For patients with BD, GM deficits were found in the medial OFC, inferior temporal and fusiform regions, insular cortex, hippocampus, and cerebellum (Figure 1 and Supplementary Table 1). When compared TIV among three patient groups, we found the significant difference between SZ and BD, (*t* = − 2.184, *p* = 0.029), BD and DP (*t* = 2.009, *p* = 0.047), and the TIV, there was no significant difference between SZ and DP (*t* = 0.143, *p* = 0.886).

**FIGURE 2:**
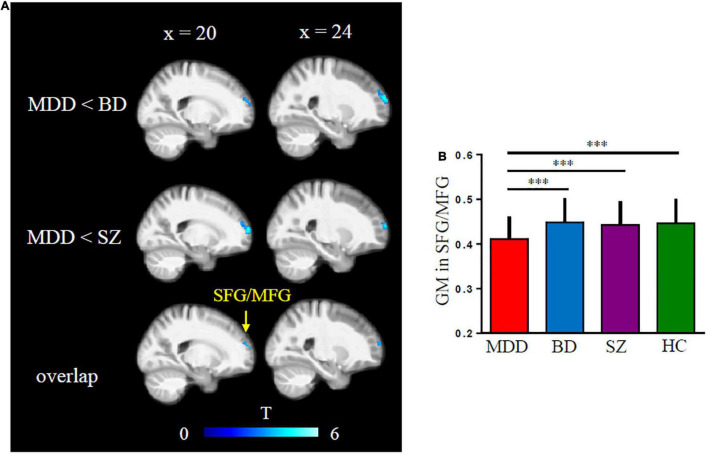
Major depressive disorder (MDD) showed reduced gray matter (GM) volumes in superior and middle frontal gyri (SFG/MFG) compared with bipolar disorder (BD) and schizophrenia (SZ) patients **(A,B)**.

**FIGURE 3:**
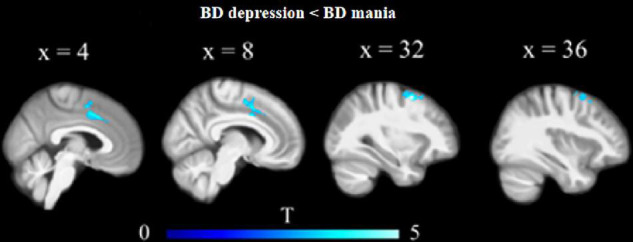
Reduced GM volumes in the middle cingulum and middle frontal gyrus in the BD depression subgroup when compared with the BD mania subgroup.

Orbitofrontal cortex GM sizes were correlated with processing speed as assessed with the DSST across four groups and PANSS positive scales only in patients with SZ (*r* = 0.17 and − 0.27, both *p* < 0.03; Figure 1). In addition, patients with MDD had significantly smaller GM in the superior and middle frontal gyri (SFG/MFG; Figure 2). However, GM sizes in SFG/MFG were not significantly correlated with BAI in patients with MDD and BD (*r* = − 0.10, *p* = 0.360), or in MDD (*r* = 0.11, *p* = 0.301) or BD (*r* = − 0.13, *p* = 0.419) patients only. We also found reduced GMVs in the middle cingulum and middle frontal gyrus in the BD depression subgroup when compared with the BD mania subgroup (Figure 3).

Further, we also found common changes in GM size in the insular cortex in MDD and BD, in the frontal lobe, temporal pole, and occipital lobe in MDD and SZ, and in the hippocampus and cerebellum in BD and SZ.

## Discussion

In this study, we revealed a transdiagnostic feature of GMV decrease in the OFC across SZ, MDD, and BD. Meanwhile, the specific GMV was decreased in the frontal and temporal lobes, insular cortex, and occipital regions in MDD, in the medial OFC, inferior temporal and fusiform regions, insular cortex, hippocampus, and cerebellum in BD, and in the frontal lobe, temporal pole, occipital lobe, thalamus, hippocampus, and cerebellum in SZ. The present study further provides evidence for the common and specific GMV loss in SZ, MDD, and BD.

The present study is a part of the systematic exploration of the common and specific MRI features of SZ, MDD, and BD in the Chinese population. Our published studies revealed a decreased functional connectivity in the insula (Yang et al., 2019) and reduced white matter integrity in the body and genu of the corpus callosum and corona radiata across these three psychiatric disorders (Cui et al., 2020). The corpus callosum and corona radiata are related to the prefrontal cortex (PFC) and ACC (Goodkind et al., 2015; Dong et al., 2017), and the corona radiata is also related to the pathway of the anterior insula (Nomi et al., 2018). The previous meta-analysis reported common GM loss in the dorsal ACC, right insula, and left insula across six psychiatric disorders that include SZ, MDD, and BD (Goodkind et al., 2015). Meanwhile, a study in the Chinese population found that common GM was decreased in the OFC, dorsolateral PFC, angular gyri, cingulate gyri, parahippocampal gyri, and temporal pole (Chang et al., 2018). Recent meta-analysis found that the decreased GM in the right cerebellum might be a common brain structural abnormality across SZ, BD, and MDD, and the regional GM abnormalities in thalamus, neocortex, and striatum appear to be disorder-specific (Zhang et al., 2020). Interestingly, our finding suggested that the GMV decreased in the OFC, which is part of the PFC, is a common feature of the three psychiatric disorders. Therefore, our finding is consistent with a previous study (Chang et al., 2018) and further supported by the loss of GMV in psychiatric disorders (Goodkind et al., 2015; Chang et al., 2018). Therefore, our findings indicated that OFC impairment may be correlated with brain function in MDD, SZ, and BD. Previous functional connectivity studies have reported that the default mode network (DMN) plays an important role in psychiatric disorders (Meda et al., 2014; Cheng et al., 2022; Pang et al., 2022). Since the OFC is located in the DMN, further research needs to explore the functional abnormalities of the OFC in these three psychiatric disorders.

Orbitofrontal cortex, one of the three main regions of the PFC, plays a role in affective and cognitive processes, such as the integration of multiple sensory information (Forbes and Grafman, 2010). The OFC has been implicated in MDD (Bremner et al., 2002), BD (Konarski et al., 2008), and SZ (Haijma et al., 2013) in previous studies. A previous study found decreased GMVs in the OFC, dorsolateral PFC, insula, temporal pole, cingulate gyri, parahippocampal gyri, and angular gyri across SZ, MDD, and BD (Chang et al., 2018). The present study is consistent with this study, as we found a decreased GMV of the OFC in these three psychiatric disorders. In the present study, 65.9% of all patients had a recurrent episode and had a long illness duration, whereas, in previous studies, 72.9% (Chang et al., 2018) and 67.8% (Xia et al., 2018) of all patients had the first episode and short illness duration. Meanwhile, the common genetic variations are 15% for SZ and BD, 10% for MDD and BD, and 9% for MDD and SZ (Lee et al., 2013). This may explain why our findings with respect to morphology in these three psychiatric disorders were different from those of a previous study on six psychiatric disorders (Goodkind et al., 2015).

Previous studies have confirmed that the insula plays an important role in patients with depression or BD, both from structural (Wise et al., 2016) and functional imaging (Wang et al., 2019), and may be a potential biomarker. In the present study, we found a common decrease in GMV in the insular in MDD and BD. This was in line with previous studies (Bora et al., 2012; Wise et al., 2016). Further, common decreases in GMV between SZ and BD were observed in the hippocampus and cerebellum, which is inconsistent with the common decreased GMV in the STG and inferior parietal lobule (Cui et al., 2011). Meanwhile, our study provided further evidence that the GMV was decreased in the frontal lobe, temporal pole, and occipital lobe in MDD and SZ.

Specifically, we revealed GM deficits in the frontal and temporal lobes, insular cortex, and occipital regions in MDD. These results are partly supported by a previous study, which reported that the GMV was reduced in the lateral temporal and occipital cortices in MDD (Frodl et al., 2008). One study also found that GM decreases in the right precentral gyrus in BD (Chang et al., 2018). Further, our study is in line with the founding of GM deficits in the bilateral insular cortex in BD (Wise et al., 2016). Meanwhile, specific GM deficits in the temporal pole (Colibazzi et al., 2017), thalamus (Anticevic et al., 2015; Zhang et al., 2020), PFC, and hippocampus (Ganzola et al., 2014) in SZ were also in consistence with our findings. Therefore, those specific reduced GMV brain regions may provide objective biological markers for image diagnosis and discriminate SZ, MDD, and BD.

A recent study found the associations between psychopathological syndromes and regional GMV across affective and psychotic disorders. Especially found positive formal thought disorder was correlated with the GMV of bilateral OFC (Stein et al., 2021). There was also a follow-up study in SZ that showed the important role of OFC in SZ. They found that greater pre-treatment OFC GMV was associated with greater post-treatment improvement in positive symptoms, particularly in hallucinations and persecutory beliefs (Premkumar et al., 2015). Therefore, our result was obtained at baseline MRI and also supports that the decreased GMV of OFC was correlated with positive symptoms in SZ. Executive function is related to the fronto-cingulo-parietal network (van Amelsvoort and Hernaus, 2016) and includes basic cognitive processes, such as attentional control, working memory, cognitive inhibition, and flexibility. It is impaired in multiple disorders, such as MDD, BD, and SZ (Hosenbocus and Chahal, 2012; Etkin et al., 2013). The OFC is related to the visual cortex, and decreased GM of the visual cortex is significantly associated with poor executive function (Goodkind et al., 2015). In the present study, we found that the OFC was correlated with processing speed as assessed with the DSST. Meanwhile, processing speed is considered as a part of cognitive processes. Therefore, our study suggested that OFC impairment may be related to executive function deficits.

Our study had some limitations. Firstly, compared with a previous study (Chang et al., 2018), the sample size of our BD patient group was relatively small. Future studies of the Chinese population will need to include multi-site samples to enlarge the sample sizes. Secondly, factors, such as duration of illness and age of onset, are difficult to control and match between the three groups. Previous research (Chang et al., 2018) was also difficult to achieve such an ideal state and reported that there had significant differences in duration (months), and the first episode (years) among SZ, MDD, and BD. Finally, most patients in the present study were treated with medication. Of 197 patients, 147 (74.6%) used at least one type of medication, such as antipsychotics, antidepressants, and mood stabilizers. The effects of the medication may have influenced the results. However, a recent study reported no significant difference in SZ, MDD, and BD between patients with and without medication (Chang et al., 2018). Moreover, data on medication-free patients are not easily available. Future studies will be needed to analyze the effects of medication.

## Conclusion

In conclusion, our findings of common OFC alterations in SZ, MDD, and BD provided evidence that this region dysregulation may play a critical role in the pathophysiology of these three psychiatric disorders. In addition, our findings indicated that based on reduced GMV in specific regions, we were able to discriminate MDD from SZ and BD, SZ from MDD and BD, or BD from MDD and SZ. Future studies will need to make a verification for these findings and evaluate the common and specific connection features at the brain network level and in different ethnic groups.

## Data Availability Statement

The datasets presented in this article are not readily available because the datasets generated and/or analyzed during the current study are not publicly available due to privacy. Requests to access the datasets should be directed to corresponding authors.

## Ethics Statement

The studies involving human participants were reviewed and approved by the Ethics Committee of the Second Affiliated Hospital of Xinxiang Medical University. The patients/participants provided their written informed consent to participate in this study. Written informed consent was obtained from the individual(s) for the publication of any potentially identifiable images or data included in this article.

## Author Contributions

LL and JS designed the study protocol. YY, YL, and YZ conducted sample selection and data management. YY, WL, KL, and YC managed the literature searches and analysis. YY, YC, LZ, and XL wrote the first draft of the manuscript. All authors contributed to and have approved the final manuscript.

## Conflict of Interest

The authors declare that the research was conducted in the absence of any commercial or financial relationships that could be construed as a potential conflict of interest.

## Publisher’s Note

All claims expressed in this article are solely those of the authors and do not necessarily represent those of their affiliated organizations, or those of the publisher, the editors and the reviewers. Any product that may be evaluated in this article, or claim that may be made by its manufacturer, is not guaranteed or endorsed by the publisher.

## Footnotes

1
http://www.fil.ion.ucl.ac.uk/spm/
