# Supplementary material for: Reduced Gray Matter Volume in Orbitofrontal Cortex Across Schizophrenia, Major Depressive Disorder, and Bipolar Disorder: A Comparative Imaging Study
Source: Front Neurosci. 2022 Jun 10;16:919272. doi: 10.3389/fnins.2022.919272 (PMC9226907; doi:10.3389/fnins.2022.919272)
Supplement: Supplementary file 1 [file Table_1.DOCX]

**Supplementary Table 1** Common and specific gray matter volume reductions in SZ, MDD, and BD brain regions.

| Group | Region | Cluster size | Peak MNI coordinates(mm) | | | t value |
| --- | --- | --- | --- | --- | --- | --- |
|  |  | (voxels) | x | y | z |  |
| SZ | Frontal Lobe | 603 | -13.5 | 22.5 | -22.5 | 3.71 |
|  | Temporal Lobe | 398 | -52.5 | -51 | -16.5 | 4.85 |
|  | Occipital Lobe | 715 | 4.5 | -91.5 | 22.5 | 3.72 |
|  | Thalamus | 124 | 57 | -10.5 | 10.5 | 5.02 |
|  | Hippocampus | 436 | 57 | -10.5 | 10.5 | 5.02 |
|  | Cerebellum | 1123 | 57 | -10.5 | 10.5 | 5.02 |
| MDD | Frontal Lobe | 555 | -34.5 | 31.5 | 34.5 | 3.37 |
|  | Temporal Lobe | 590 | -48 | -1.5 | -18 | 3.39 |
|  | Occipital Lobe | 609 | 3 | -73.5 | -6 | 4.25 |
|  | Insular Cortex | 915 | -54 | 3 | 1.5 | 5.36 |
| BD | Medial Orbitofrontal Cortex | 159 | 7.5 | 39 | -21 | 4.38 |
|  | Inferior Temporal Gyrus | 207 | 58.5 | -48 | -16.5 | 4.00 |
|  | Fusiform Gyrus | 209 | -25.5 | -60 | -13.5 | 4.47 |
|  | Insular Cortex | 156 | 30 | 30 | 0 | 3.49 |
|  | Hippocampus | 45 | 37.5 | -30 | -9 | 3.62 |
|  | Right Cerebellum | 489 | 37.5 | -45 | -37.5 | 3.82 |
| Shared regions | Orbitofrontal Cortex | 96 | 3 | 39 | -13.5 | 3.53 |

SZ, schizophrenia; MDD, Major Depressive Disorder; BD, Bipolar Disorder; MNI, Montreal Neurological Institute.
